# History of narcolepsy at Stanford University

**DOI:** 10.1007/s12026-014-8513-4

**Published:** 2014-05-14

**Authors:** Emmanuel J. M. Mignot

**Affiliations:** Stanford University Center for Sleep Sciences, 3165 Porter Drive, #2178, Palo Alto, CA 94304 USA

**Keywords:** Narcolepsy, Cataplexy, HLA, MHC, DQB1*06:02, Autoimmune disease

## Abstract

Although narcolepsy was first described in the late nineteenth century in Germany and France, much of the research on this disorder has been conducted at Stanford University, starting with Drs. William C. Dement and Christian Guilleminault in the 1970s. The prevalence of narcolepsy was established, and a canine model discovered. Following the finding in Japan that almost all patients with narcolepsy carry a specific HLA subtype, HLA-DR2, Hugh Mac Devitt, F. Carl Grumet, and Larry Steinman initiated immunological studies, but results were generally negative. Using the narcoleptic canines, Dr. Nishino and I established that stimulants increased wakefulness by stimulating dopaminergic transmission while antidepressants suppress cataplexy via adrenergic reuptake inhibition. A linkage study was initiated with Dr. Grumet in 1988, and after 10 years of work, the canine narcolepsy gene was cloned by in 1999 and identified as the hypocretin (orexin) receptor 2. In 1992, studying African Americans, we also found that DQ0602 rather than DR2 was a better marker for narcolepsy across all ethnic groups. In 2000, Dr. Nishino and I, in collaboration with Dr. Lammers in the Netherlands, found that hypocretin 1 levels in the cerebrospinal fluid (CSF) were undetectable in most cases, establishing hypocretin deficiency as the cause of narcolepsy. Pursuing this research, our and Dr. Siegel’s group, examining postmortem brains, found that the decreased CSF hypocretin 1 was secondary to the loss the 70,000 neurons producing hypocretin in the hypothalamus. This finding revived the autoimmune hypothesis but attempts at demonstrating immune targeting of hypocretin cells failed until 2013. At this date, Dr. Elisabeth Mellins and I discovered that narcolepsy is characterized by the presence of autoreactive CD4^+^ T cells to hypocretin fragments when presented by DQ0602. Following reports that narcolepsy cases were triggered by vaccinations and infections against influenza A 2009 pH1N1, a new pandemic strain that erupted in 2009, our groups also established that a small epitope of pH1N1 resembles hypocretin and is likely involved in molecular mimicry. Although much remains to be done, these achievements, establishing hypocretin deficiency as the cause of narcolepsy, demonstrating its autoimmune basis, and showing molecular mimicry between hypocretin and sequences derived from a pandemic strain of influenza, are likely to remain classics in human immunology.

## Early years

Starting in the late nineteenth century, narcolepsy has been recognized as a unique syndrome distinct from epilepsy [1]. It was shown to include severe daytime sleepiness and cataplexy, defined as sudden episodes of muscle weakness triggered by emotions, typically laughing or joking. The first case reports in a bookbinder and a cooper (barrel maker and seller), published by Westphal in 1877 [2] and Gelineau in 1880 [3], are still strikingly similar to cases we see today, although onset was late (in adulthood) unlike most cases we see today (childhood and adolescent). As an example of cataplexy, Gelineau’s patient reported collapsing at the Zoo of the “Jardin des Plantes,” while observing monkeys making faces. Symptoms recognized soon after included sleep paralysis, vivid dreaming, hypnagogic hallucinations, disturbed nocturnal sleep, and weight gain [4–6]. When cataplexy was present, most clinicians were of the opinion the syndrome was a discrete disease entity with a specific pathophysiology [7], something time has proven to be correct, as cataplexy is the best predictor of an absence of hypocretin in the cerebrospinal fluid (CSF) of patients with narcolepsy [8]. Table 1 reports on the most important milestones of narcolepsy research from these early years to today.

**Table 1:** A few milestones in narcolepsy research and therapy

1877	First description in the medical literature [1, 2]
1880	Gelineau called the disorder “narcolepsy” [1, 3]
1902	Loewenfeld coined the term “cataplexy” [215]
1935	First use of amphetamines in the treatment for narcolepsy [216]
1960	Description of sleep onset REM periods in a narcoleptic subject [19–21]
1973	First report of a narcoleptic dog [61, 217]
1977	Description of the multiple sleep latency test [30, 31]
1983	Association of narcolepsy with HLA-DR2 [33, 34, 218]
1985	Monoaminergic and cholinergic imbalance in narcolepsy [62, 68]
1992	Association of narcolepsy with HLA-DQB1*06:02 [56, 60]
1998	Identification of hypocretins/orexins and their receptors [18, 93, 105, 106]
1999	Hypocretin system mutations cause narcolepsy in mice and dogs [104, 120]
2000	Human narcolepsy is also associated with low CSF hypocretin 1 [8, 123]
2000	Human narcolepsy is associated with a loss of hypocretin cells [121, 124, 126]
2001	HLA effects additional to DQB1*06:02 in narcolepsy [146]
2009	Genome-wide associations identify T-cell receptor as additional susceptibility loci [154–156, 204]
2010	Association of narcolepsy onsets with 2009 H1N1 infections and vaccinations [143, 178, 184–186]
2010	Allele competition explains protective effects of some DQ1 haplotypes [95]
2013	Identification of hypocretin-autoreactive CD4^+^T cells in narcolepsy [198]
2013	Molecular mimicry between H1N1 and hypocretin autoantigen [198]

## Flu-hypothalamic connection and Von Economo’s visionary work

Although the cause of narcolepsy, a loss of hypocretin cell in the hypothalamus, was not known until recently, insight came from the work of Constantin Von Economo [9]. Constantin Van Economo was a true European before its time. Of Greek-Macedonian descent, he was born in Brăila, now Romania in 1876 and raised in Trieste, now Italy. As citizen of the Austro-Hungarian Empire, he was sent to Vienna to study mechanical engineering, but rapidly switched to medicine. Travelling across Europe and studying with the best, he finally settled back in Vienna at the Clinic for Psychiatry and Nervous Diseases.

In the 1918–1923, immediately following the 1918 devastating Spanish flu, an H1N1 flu epidemic that killed over 50 million individuals [10], another seasonal epidemic occurred where patients had severe encephalitis, with high mortality rates and significant brain pathology including “non-purulent, non-hemorrhagic acute inflammation on the gray matter.” Lymphocytic infiltrates, also often perivascular, are noted with edema and occasional area of necrosis (lesions described as not unlike in poliomyelitis). A major symptom of the disease was extreme sleepiness (thus the name encephalitis lethargica), a symptom often associated with ophthalmoplegia. Other presentations of the disease included insomnia or reversal of the sleep/wake cycle, movement disorders, and psychiatric symptoms. Following the somnolent-ophthalmoplegic form, many subjects improved but had residual Parkinson’s disease [9].

That the encephalitis lethargica epidemic subsided after a few years, and that it occurred so soon after the 1918 flu epidemic, has long suggested that the epidemic was connected to the flu, although this has been highly debated. A similar secondary epidemic “Noma” was reported the years following the 1889–1890 epidemic flu in Italy [9]. Further, more encephalitis lethargica cases were reported in the western (New Zealand) versus eastern (United States) Samoan Islands, where a stricter quarantine against the 1918 flu was observed [11]. Others have however recently suggested that the epidemic was due to another, unrelated virus, with some recent pathophysiological support in favor of an enterovirus [12]. As is often the case, the passage of time, and a lack of samples are the worst enemies of investigation, making it difficult to determine what happened.

**Fig. 1:**
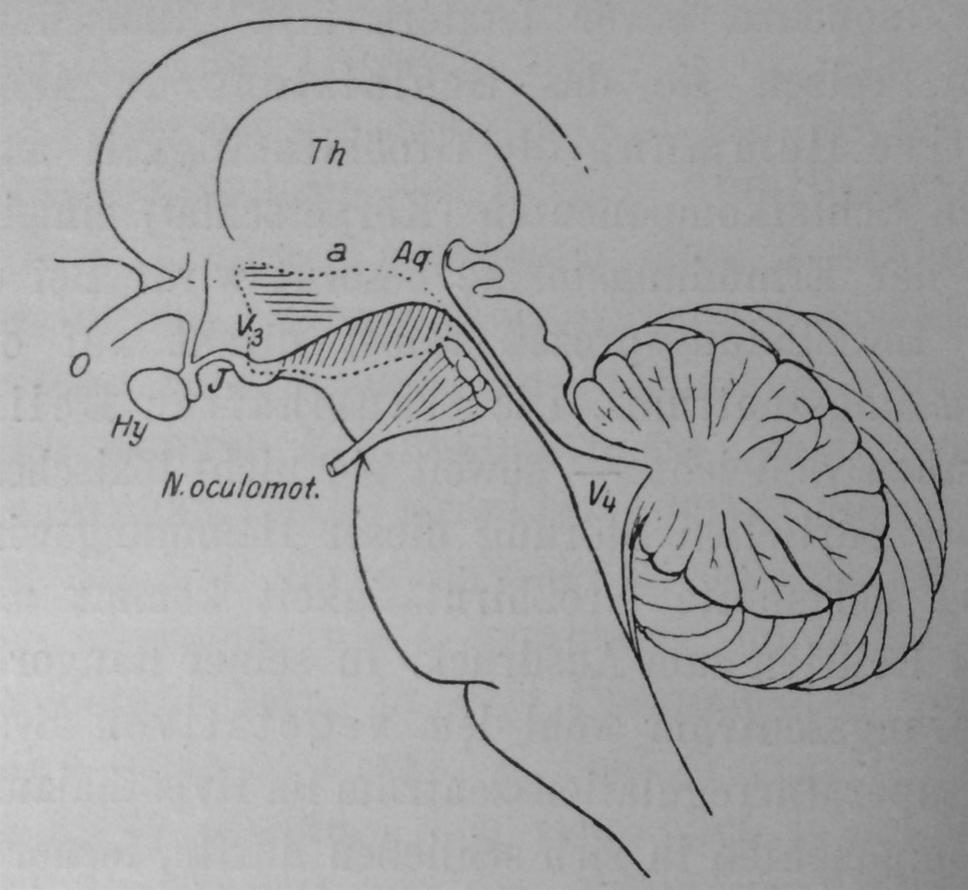
Van Economo’s sleep regulatory centers, marked by the *dotted line* in the transitional region from the diencephalon to the mesencephalon. *Aq* aqueduct, *Hy* hypophysis, *J* infundibulum, *N.* occulomot.: third cranial nerve. *O* optic chiasm, *Th* thalamus, *V3* and *V4* ventricles. Marked by *parallel oblique vertical lines* (posterior hypothalamus and upper brainstem): region whose affection produces sleep; marked by *horizontal lines* (anterior hypothalamic regions): region whose affection produces insomnia from Van Economo [9]

Insights into the pathophysiology of narcolepsy came from clinico-pathophysiological studies of these cases. Indeed, Von Economo [9], studying postmortem samples and correlating symptoms with areas of necrosis or cellular infiltrations in the brain, noticed that the best correlation with sleepiness was damage to the posterior hypothalamic area, extending to the upper pons and the third cranial nerve (Fig. 1), a region known now to contain most essential wake-promoting systems, including hypocretinergic systems causal of narcolepsy–cataplexy, but also wake-on monoaminergic systems such as the histaminergic tuberomammillary nucleus and other important sleep-regulatory systems [13].

Importantly, however, only a handful of encephalitis lethargica cases had cataplexy [7, 14], the hallmark of narcolepsy, although atypical atonia could have been missed in the context of the more complex clinical picture. Other clinico-anatomical correlations made by Von Economo included a correlation between damage in the anterior preoptic hypothalamus with insomnia (a region known now to contain preoptic sleep-promoting GABAergic systems) [9]. Other investigators had noted prior that cases of secondary narcolepsy were often associated with tumors located close to the third ventricle [7, 15].

## Sleep onset REM sleep as a feature of narcolepsy

The discovery of rapid eye movement (REM) sleep by Aserenski and Kleitman in Chicago in 1953 opened the area of modern sleep research [16]. In parallel with this work, Jouvet described “paradoxical sleep” pointing out that a pervasive atonia with brief bursts of phasic activity was present during this stage of sleep [17]. William C Dement, who trained as a psychiatrist and was a graduate student in Kleitman’s laboratory when REM sleep was discovered, became interested in dreaming and reported the common association of this phenomenon with REM sleep [18]. From these observations and the clinical descriptions of narcolepsy, it became quickly evident that narcolepsy involved abnormal REM sleep. Working with Alan Rechschaffen, Dement described that unlike controls who typically entered their first REM sleep period 90 min after sleep onset, patients with narcolepsy often went directly into REM sleep during nighttime sleep testing, a phenomenon we call sleep onset REM periods (SOREMPs) [19, 20]. A similar finding was also reported by Vogel et al. [21]. Subsequent studies, still valid today, found that only 50 % of cases entered REM sleep within 15 min of sleep onset during nocturnal sleep studies, limiting its usefulness as a clinical test [22].

## The Stanford Sleep Clinic and first narcolepsy prevalence studies

William C. Dement joined Stanford University in 1963 [23, 24]. Seeking narcoleptic subjects for his studies, he conducted one of the first prevalence studies for the condition and also started a small sleep clinic to see these patients in 1964. He identified many patients within the San Francisco Bay area using newspaper advertisements and a description of the syndrome [25]. By considering the number of cases that responded to the advertisement and readerships of the add, he estimated the prevalence at 0.07 %, a figure remarkably similar to the currently accepted prevalence of 0.03–0.05 %, established through dozens of well-designed population-based studies across the world [26, 27]. Dement was surprised by the unexpectedly high frequency and saw many patients as the result of this study, most of whom discovered their condition thanks to the advertisement. However, population size was not sufficient to support a narcolepsy-only clinic, and clinical activity stopped in 1965. In 1970, Dr. Christian Guilleminault joined the clinic with a primary interest in sleep-disordered breathing and coined the term obstructive sleep apnea [23, 24, 28]. Sustained clinical activity resulted, and the Stanford Sleep Clinic became a beacon for the field, eventually leading to the establishment of sleep medicine as a distinct medical specialty [29].

The new Sleep Disorders Clinic introduced all-night polysomnographic examination of patients with sleep-related complaints, medical responsibility and management of the patient, and objective assessment of the relationship between nighttime sleep and daytime function. For the latter, Dement and Carskadon developed the multiple sleep latency test (MSLT), which remains the standard diagnostic measure of daytime sleepiness [23, 30]. In this test, patients or volunteers are asked to nap every 2 h (while staying awake in between) and the mean sleep latency measured as an objective measure of daytime sleepiness. Using the MSLT, Richarsdon, Mitler, and others found that narcoleptic patients often have naps containing REM sleep in contrast to controls [31, 32]. They found that at least two SOREMP and a short mean sleep latency is a reliable objective test for narcolepsy [31, 32]. Together with the demonstration of REM sleep onset during nocturnal sleep [22], the MSLT is still the most commonly used diagnostic test for narcolepsy.

## First HLA association studies in narcolepsy and early immunological studies

In 1983, as part of a program to search for HLA associations in orphan disorders, a weak association was recognized with HLA-Bw35 in Japanese patients [33]. At the time, HLA type was defined using panels of autologous antibodies and not molecular typing. Following on these findings, Juji and Honda found that 100 % of Japanese narcolepsy–cataplexy patients carried HLA-DR2 and DQ1 versus 25 % of controls [34, 35]. This was rapidly confirmed across the world [36–39], including at Stanford University [40], where nonetheless very rare DR2-negative cases were identified, causing controversy between the Stanford group and Japan [35, 40, 41]. Current results indicate that although the association between hypocretin deficiency and HLA is extremely high (98 %), rare exceptions have been documented (see below).

The result was nonetheless remarkable: over 95 % of cases with cataplexy carried DR2, DQ1 versus 25 % in general Caucasian and Japanese populations [37, 42]. Using another technique, mixed leukocyte culture (MLC), DR2 antigens were found to be heterogeneous, including Dw12, Dw2, Dw21, and Dw22 subtypes [43, 44]. All Japanese patients carried Dw2, which is the dominant DR2 subtype in Caucasians (25 %), but a more minor antigen in Japanese (8–10 %), where Dw12 is more common [44, 45]. Restriction fragment length polymorphism (RFLP) studies with DQ probes performed in the laboratory of Hugh McDevitt also confirmed this finding, showing a unique pattern of association correlating with Dw2 but not Dw12 [46, 47]. As will be seen later, this pattern differentiates DRB1*15:01, DQA1*01:02, DQB1*06:02 versus DRB1*15:02, DQA1*01:03, DQB1*06:01, the two major DR2 and DQ1 haplotypes in the Japanese population [48].

The HLA genes, also called major histocompatibility (MHC) genes, are located on human chromosome 6. As noted by Hugh McDevitt [49] at Stanford, MHC polymorphisms are essential contributors to genetic diversity in the immune response, allowing more diverse epitope presentation across individuals. Although HLA polymorphisms modulate immune responses to infections, these polymorphisms were rapidly shown to be most strongly associated with autoimmune diseases [50]. Considering this hypothesis for narcolepsy, researchers studied inflammation around disease onset [51], or tried to identify brain-specific autoantibodies [52], but found no evidence for autoimmunity [53, 54]. As all results were consistently negative, researchers speculated that HLA DR2 was perhaps only a linkage marker for a yet unknown sleep gene located in this region of the genome. As genetic mapping and characterization of the HLA region progressed, this second hypothesis became less and less likely [55]. As described below, in 1992–1997, studies demonstrated clearly that the closely linked HLA-DQB1*06:02 and DQA1*01:02 loci (forming the DQ0602 heterodimer) rather than DR2 was the best marker for narcolepsy, and that the association signal rapidly decreased on both sides of the HLA-DQ locus [56–58]. Sequencing of these HLA genes also showed no abnormalities [59, 60].

## Canine narcolepsy

In 1972, Dement presented video recordings of patients with narcolepsy–cataplexy at an educational exhibit during the annual convention of the American Medical Association in San Francisco [24]. A member of the audience, on the veterinary faculty at University of California Davis, noticed the resemblance with a canine patient he had seen with a provisional diagnosis of refractory epilepsy. Unfortunately, the dog had been euthanized, but a video was available, showing the similarity with human narcolepsy, with the dog collapsing but awake when excited by food or other activities. After a national search and contacting veterinarians across North America, “Monique,” a French poodle was identified and donated to Stanford [61]. In the following few years, Drs. Dement and Mitler visited veterinarians in more than 50 cities and spoke at many colleges of veterinary medicine in the United States. Small numbers of animals of various breeds with narcolepsy were identified, and a small canine colony was established [62]. Poodles and Beagles were bred (backcrosses included) but genetic transmission was not established in these breeds [62, 63], later shown to have hypocretin deficiency [64].

**Fig. 2:**
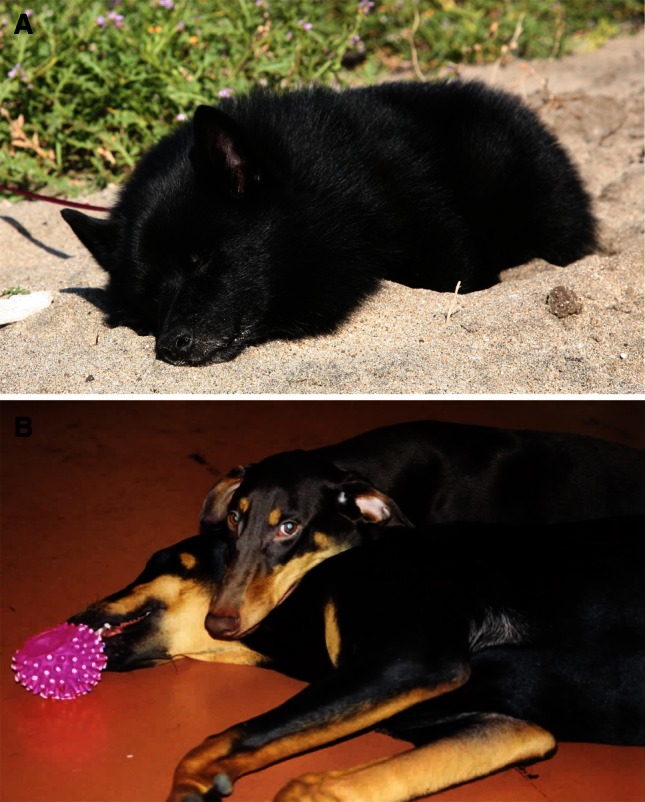
Narcoleptic dogs of the colony, a sporadic case with hypocretin deficiency (**a**) and two familial cases with hypocretin receptor 2 mutations (**b**). The dog on the *left* was later shown to have low CSF hypocretin, like human narcolepsy [64]. The bottom dog on the *right* is having a complete attack of cataplexy, an episode of complete muscle paralysis while awake that has been triggered by the excitement of playing with his littermate

In 1975, two affected Doberman littermates and one unrelated Doberman with narcolepsy were donated to the colony [62, 63]. Breeding these animals led to the first successful genetic transmission of narcolepsy, with a litter of affected animals born at Stanford on July 29, 1976. Multiple cases of Labradors with narcolepsy were subsequently reported and, with the help of Dr. Cavalli-Sforza, the trait found to be transmitted as a single autosomal recessive gene [62, 63]. Canine narcolepsy (Fig. 2) was characterized in detail at the clinical level [65–69] but many investigators refused to believe these animals had narcolepsy.

In the late 1980s, following the HLA discovery in humans [34], efforts were made to see whether a similar MHC association could be found in canine narcolepsy, whether the genetic form or the sporadic form. Studies using mixed leukocyte culture [70] and RFLPs in canine narcolepsy did not identify shared MHC DR or DQ genes [71], as in humans. Interestingly, whereas the lack of association in autosomal recessive narcolepsy is not surprising considering its pathophysiology (mutations in the hypocretin receptor 2, see below), it is still unclear why sporadic canine narcolepsy, a disorder commonly associated with low CSF hypocretin as in humans [64], does not show a clear MHC DR and DQ association.

## Neurochemical and pharmacological studies in canine narcolepsy suggest a downstream imbalance of monoaminergic and cholinergic systems, and dysregulated dopaminergic transmission in the amygdala

In the 1970s, the study of monoaminergic and cholinergic transmission in the regulation of sleep emerged as a leading research avenue [72]. Thanks to pioneering transection experiments by Jouvet [17], the pons was considered as a logical first candidate region for a narcolepsy abnormality. The levels of acetylcholine, various monoamines, metabolites, and receptors were measured in the cerebrospinal fluid and various brain regions of narcoleptic animals and humans [73–81]. These led to the general hypothesis of a pontine monoaminergic–cholinergic imbalance in narcolepsy [62]. In this model, narcolepsy was the result of cholinergic hyperactivity and monoaminergic hypoactivity in the pons, a concept paralleling the then fashionable Hobson and McCarley model for REM sleep regulation [82, 83]. A primary role of the amygdala was also proposed, based on the observation of consistent dopaminergic abnormalities in this brain region [80, 81]. The involvement of this structure is attractive conceptually, as it may explain why cataplexy is triggered by emotions.

Working with Tom Kilduff, Craig Heller, and others, Dr. Nishino and I next conducted a systematic pharmacological dissection of the mode of action of the then commonly prescribed narcolepsy treatments, stimulants for sleepiness, and antidepressants for cataplexy [68]. This led us to demonstrate that presynaptic activation of adrenergic transmission mediates the anticataplectic effects of antidepressants [84, 85], a finding that still has application today, as serotonin norepinephrin reuptake inhibitors (SNRI) such as venlafaxine or pure adrenergic reuptake inhibitors, such as atomoxetine, are still used to treat human cataplexy [5]. We also established that wake-promoting effects of amphetamine stimulants and modafinil are mediated by a presynaptic activation of dopaminergic transmission [85–87]. The idea that modafinil was acting through dopaminergic reuptake inhibition was contested by Lafon Laboratories of France (the original inventor of modafinil) and then Cephalon (the company which owned modafinil until recently) and most other scientists, until recent studies by Volkow et al. confirmed our data in vivo [88]. Other studies emphasized the importance of cholinergic hypersensitivity in the basal forebrain area [89], mesolimbic dopaminergic hypoactivity [90] as important pathophysiological abnormalities in canine narcolepsy. Although these data were informative and documented a large overlap between REM sleep regulation and narcolepsy pathophysiology, it was not moving us forward the true cause of the condition.

## Transethnic studies demonstrate that DQ0602 is the culprit narcolepsy-associated allele

Faced with a lack of evidence for autoimmunity [53, 54], efforts focused on fine mapping the genetic association in the HLA DR region. The HLA DR and DQ region is compact, containing in sequence the DRA gene (practically monomorphic), accessory DRB genes (DRB 3, 4, 5 genes present in some but not all haplotypes), the DRB1 gene (a very polymorphic gene), an intergenic segment of about 40 kb, and finally the polymorphic DQA1 and DQB1 loci, genes separated by approximately 12 kb. Alpha genes and beta genes encode heterodimers, with the product of the DRA gene principally partnering with DRB1 to form the DRαβ molecule, while DQA1 and DQB1 encode proteins that dimerize to produce DQαβ molecules.

In Caucasians and Japanese, linkage disequilibrium between DQ and DR is so strong that practically all (>99 %) DQB1*06:02 alleles are linked with DQA1*01:02 and DRB1*15:01 (DR2) (these alleles are in full linkage disequilibrium), making it impossible to distinguish whether the effect was mediated by DR or DQ. In the early 1990s, however, studies in control African Americans revealed additional diversity in DR–DQ haplotypes, so that in this ethnic group DQB1*06:02 was found not only with DRB1*15:01, but also with DRB1*11:01 and more rarely DRB1*12:02 (all haplotypes containing DQA1*01:02) [57, 91, 92]. This, together with a study that had suggested less DR2 positivity in African American patients with narcolepsy [93], led us to test HLA-DR and DQ associations in African American controls and patients.

In 1992, to our surprise, we found that all African American patients with narcolepsy had DQA1*01:02 and DQB1*06:02 [56, 60]; however, DR was not DRB1*15 (DR2) in many instances, indicating that the primary association was with HLA-DQ, not DR, and more particularly the DQαβ heterodimer DQ0602 encoded by DQA1*01:02 and DQB1*06:02. In subsequent studies, screening hundreds of patients, we discovered a number of very rare cases of DQB1*06:02-positive subjects that carried various DRB1 alleles such as DRB1*03:01, DR8del, DRB1*08:01, DRB1*08:06, DRB1*16:01 that are only exceptionally found in controls [57, 94], confirming the enrichment of these rare DQ0602 haplotypes as well in narcolepsy. Finally, in a recent study, we found that DRB1*15:01 alone, in the context of the DRB1*15:01, DQA1*01:02, DQB1*06:01, a frequent haplotype in South China, does not predispose to narcolepsy [95]. To our knowledge, the study in 1992 was the first to take advantage of ethnic diversity in fine mapping a disease polymorphism [56, 60], something we have used to our advantage in many other subsequent studies.

## The canine narcolepsy gene is the hypocretin receptor 2

In the early part of the previous century, human narcolepsy was frequently believed to be a familial disorder, but more recent studies have shown that it is not a simple genetic disorder. Monozygotic twins are most frequently discordant for narcolepsy, indicating a requirement for environmental factors to trigger narcolepsy onset [96]. Indeed, familial clustering of narcolepsy–cataplexy is the exception rather than the rule. Only 1–2 % of the first-degree relatives of patients with narcolepsy have narcolepsy–cataplexy [96–99], although there is suggestion that as many as 4 % of first relatives have milder symptoms without cataplexy. This indicates a 20- to 40-fold increased risk when compared to the general population [96]. The complex genetics involving HLA, other genes, and environmental factors was consistent with an autoimmune basis with an unknown target.

The complex picture in human narcolepsy led us to focus our genetic studies on canines. In contrast to the human situation, narcolepsy is a simple autosomal recessive disorder in Dobermans and Labradors [63], thus making positional cloning, a technique first used in 1986 in humans, theoretically possible in this species. Backcrosses were performed and a genetic linkage study initiated in 1989 with Frank C. Grumet. Our first focus was to exclude potential candidate genes. Canine narcolepsy was shown not to be associated [70, 71] or tightly linked with dog leukocyte antigen (DLA) polymorphisms [100], suggesting canine narcolepsy gene was not an MHC gene. Additional candidate genes and minisatellite probes were used in a second stage. Using a candidate gene approach, a RFLP band cross-reacting with the human immunoglobulin µ-switch segment on a Southern blot was shown to completely cosegregate with canine narcolepsy in 1991 [100], leading to a LOD score of 7.2. This result initially suggested an immunoglobulin/immune involvement in canine narcolepsy. Further studies however demonstrated that this linkage marker was coincidentally a cross-reacting sequence of no functional significance [101].

Considering the relatively small number of animals tested, the actual narcolepsy gene was likely to be located at a large genetic distance from our initial µ-switch-like marker. Chromosome walking in the vicinity of the identified marker was difficult using available phage and cosmid genomic libraries, which have small genomic inserts making chromosome walking very slow and impractical. In 1997, Robin Li built a large insert bacterial artificial library (BAC) genomic library in collaboration with Dr. Peter De Jong [102]. The technique of fluorescence in situ hybridization (FISH) was also established in our laboratory, and the canine narcolepsy marker was found to be located on dog chromosome 12 [103], which also contains the dog leukocyte antigen (DLA) locus, but separated by a large genomic distance.

Using the newly available BAC library, we began chromosome walking in earnest. In this process, high-density gridded library filters are hybridized with DNA probes derived from BAC end sequences through the polymerase chain reaction (PCR). The new clones are then isolated, verified through PCR and FISH methods, their ends sequenced, and the filters rehybridized to extend the so-called contig of overlapping large clones. In parallel, new polymorphic linkage markers are isolated from new BAC clones by creating “mini libraries” and hybridization with microsatellite repeats [i.e., (GAAA)_n_]. These markers are then tested in canine backcrosses to confirm genetic linkage and map possible recombinant animals, which refine the map location of the mutation [104]. Also, in parallel, BAC end sequences are also analyzed using the BLAST algorithm to identify putative known genes. In 1998, an end-sequence of the BAC clone containing the μ-switch-like marker was shown to contain Myosin VI (MYO6), a gene known to map on the long arm of human chromosome 6 (6q13).

**Fig. 3:**
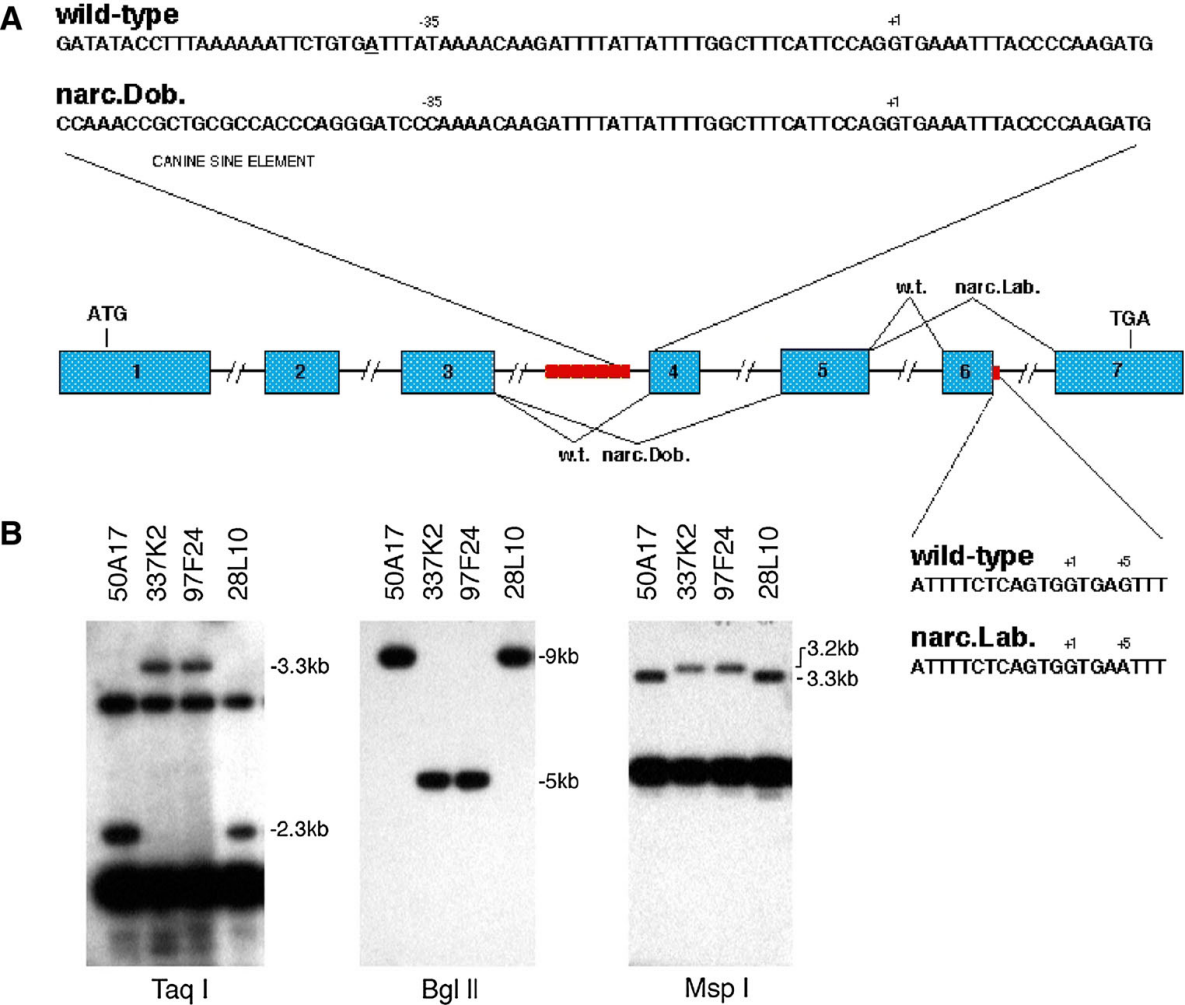
Mutations found in narcoleptic Dobermans and Labradors. **a** In Dobermans, a large SINE insertion upstream of exon4 causes exon skipping and non-functional HCRT2 receptors (Lin et al. [104] ). In Labradors with familial narcolepsy, a different mutation causes exon 6 skipping and has similar effects. **b** Western RLFP blots of BAC clones (probed with a HCRT2 human EST) derived from an heterozygous narcoleptic Doberman containing the critical region of interest. As can be seen, a different pattern is found in clones derived from narcolepsy-mutated versus control chromosomes, suggesting a significant difference surrounding HCRT2

The finding that both DLA and MYO6 were on the same dog and human chromosomes led us to suspect a large region of conserved synteny between dog chromosome 12 and human chromosome 6. This result was a turning point as it gave us direct access to the emerging human and mouse maps in the region. Human expressed sequenced tags (ESTs) known to map between MHC and MYO6 in humans were then used as probes to screen the canine BAC library. These allowed us to identify new seed BAC clones within the large critical interval from which to extend and merge our contigs. The resulting canine BAC clones were then hybridized on canine metaphase spreads to verify localization onto dog chromosome 12 [104]. Together with chromosome walking and microsatellite marker development and genetic testing in backcrosses, the process was refined until the canine narcolepsy gene was flanked in a small genetic segment known to contain only two potential genes. These two genes were tested for potential abnormalities and an abnormal RFLP hybridization pattern observed with one of the two ESTs, the hypocretin receptor 2 gene (HCRTR2) (Fig. 3) [104]. Further analysis then demonstrated that in both Labradors and Dobermans with autosomal recessive narcolepsy, the hypocretin receptor transcripts were disrupted by distinct exon splicing mutations (Fig. 3) [104]. The Ling Lin et al. [104] report was the first to implicate hypocretins/orexins in the cause of canine narcolepsy.

## Hypocretin (also called orexin) deficiency as the cause of human narcolepsy

Hypocretins/orexins were identified almost simultaneously by DeLecea et al. [105] and Sakurai et al. [106] in 1998. Luis De Lecea, Kaare Gautvick, and Tom Kilduff identified and characterized the preprohypocretin transcript (clone 35) [107] in the laboratory of Dr. Gregory Sutcliffe using a directional tag PCR subtraction technique [107]. Their aim was to identify novel hypothalamic-specific transcripts. The identified hypocretin gene was shown to be only expressed in the lateral hypothalamus and to encode a precursor molecule for two related peptides having a possible homology with secretin (this weak homology is disputed by others). Based on the selective expression of the gene in the lateral hypothalamus and their homology with the gut hormone secretin, the peptides were called hypocretins 1 and 2 by Luis DeLecea [105], who also demonstrated neuroexcitatory properties for hypocretin 2 and suggested a possible role in feeding regulation based on the neuroanatomical localization in the lateral hypothalamus [105].

The existence of hypocretins was independently confirmed by Sakurai et al. [106] in the laboratory of Masashi Yanagisawa a few weeks later. These authors also identified and mapped two receptors for these peptides (HCRTR1 and HCRTR2). In this elegant work, a series of orphan G-Protein-coupled receptors (e.g., receptor genes having no identified endogenous ligand identified) were expressed in cell lines (the “orphanage”) and the resulting cell lines used to screen high-pressure liquid chromatography (HPLC) purified tissue fractions for biological activity [106]. Cell lines containing the orphan receptor HFGAN72 (later shown to be the hypocretin receptor 1) were found to strongly react with purified brain fractions. These fractions were shown to evoke a calcium transient, suggesting the activation of the G-Protein-coupled receptor by an endogenous ligand. The resulting activity was purified and shown to be a 33 amino acid peptide that Sakurai et al. called orexin A [106]. Another weaker activity was also isolated and shown to be a 28 aminoacid peptide sharing 13/28 aminoacid identity with orexin A; this second peptide was called orexin B [106]. Both peptides were then shown to be processed from the same precursor, a transcript identical to DeLecea’s previously reported preprohypocretin mRNA molecule [105]. Hypocretins 1 and 2 and orexin A and B are thus identical with the caveat that DeLecea reported a 6 amino acid longer sequence for hypocretin 1 versus orexin A. The latter author mentioned that the N-terminal of the hypocretin 1 peptide could not yet be established at the time [105]. Sakurai et al. [106] also noted that hypocretin 2/orexin B had a lower affinity for the hypocretin receptor 1 and found that another unknown EST had high nucleotide homology with HFGAN2. This receptor was expressed in CHO cell lines and was shown to bind and mobilize calcium in the presence of hypocretins 1 and 2. This second receptor was called the orexin receptor 2 (hypocretin receptor 2 as the gene name).

The discrete localization of these peptides in the lateral hypothalamus suggested a role for hypocretins in feeding behavior. In their initial publication, Sakurai et al. [106] reported a stimulation of feeding after central administration of hypocretins/orexins and an increased preprohypocretin mRNA expression after fasting, leading them to select the name “orexin.” The authors speculated that a main physiological function for these molecules could thus be the regulation of energy homeostasis [106]. Subsequent work indicated variable effects on feeding, and more effects on metabolism and activity [108–114]. Neuroanatomical work indicating widespread projections for hypocretin neurons in the entire brain and spinal cord also suggested more complex physiological functions [115, 116]. Of note, dense projections to monoaminergic cell groups such as the locus coeruleus [117], the raphe [118], and tuberomammilary nuclei [119] suggested a possible involvement in sleep regulation. In 1999, a few weeks after canine narcolepsy was shown to be due to hypocretin receptor mutations, a knockout mouse for the preprohypocretin gene was described and shown to have sleep abnormalities reminiscent of narcolepsy [120], thus independently indicating a role for hypocretin in the sleep disorder.

The potential role of hypocretin gene mutations in human narcolepsy was almost immediately investigated by Juliette Faraco in our group [121]. Not surprisingly, considering that narcolepsy was genetically complex and likely autoimmune, mutation screening in the preprohypocretin and hypocretin receptor genes yielded few positive results. In only one case was a highly suspicious mutation found, a L > R polar amino acid substitution in the hydrophobic polyleucine track of the signal peptide. The clinical picture was very atypical, combining undetectable CSF hypocretin 1, DQ0602 negativity, and very early onset at 6 months of age [122]. Unfortunately, the father DNA was not available to confirm a de novo mutation, although transfection studies of the mutant allele in neural cells revealed abnormal processing/trafficking, with elaboration of a tubular-like cellular compartment and resulting toxicity, explaining the likely dominant phenotype. No mouse model was ever generated to fully confirm this hypothesis.

In parallel with this work, hypocretin 1 levels were first measured in the cerebrospinal fluid (CSF) of 9 narcoleptic subjects and 8 controls by Nishino et al. [123]. Seven narcoleptic subjects were found to have undetectable hypocretin 1 levels. Two narcoleptic patients had normal levels of hypocretin 1, respectively. Hypocretin 1 levels were detectable in all controls. This result suggested that human narcolepsy was caused by a deficiency in hypocretin production [123]. A simple explanation was that hypocretin-producing cells are destroyed by an autoimmune process in HLA-associated narcolepsy. Only hundred thousand cells in the hypothalamus produce these peptides, and a discrete lesion in this area might not have been detected in previous neuropathological studies.

**Fig. 4:**
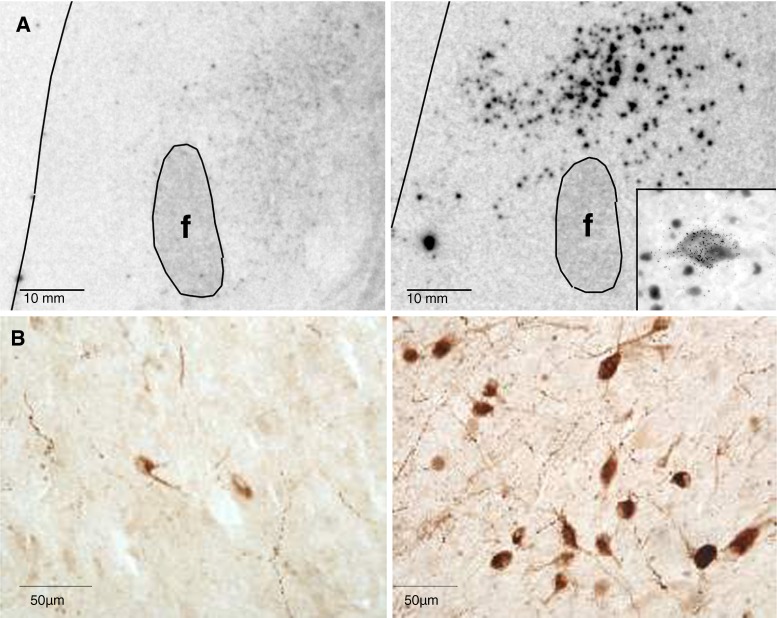
Hypocretin cell loss as the cause of narcolepsy, as demonstrated by in situ hybridization (**a**), from Peyron et al. [121], *f* fornix) and hypocretin peptide immunocytochemistry (**b**), from Thannickal et al. [124] ). *Right* narcolepsy samples, *left* control brains

Indeed, two studies were quickly published demonstrating the loss of hypocretinergic cells in human narcolepsy brain tissue, supporting this hypothesis. In one study performed by Christelle Peyron, in situ hybridization of the perifornical hypothalamus and peptide radioimmunoassay measurements in six human brains indicated a global loss of hypocretins, without signs of inflammation in all human cases examined [121] (Fig. 4). The second study, published a few weeks later, used immunocytochemistry and found a 85–95 % reduction in the number of hypocretin neurons in 4 narcolepsy brains (one without cataplexy) [124]. In both studies, melanin-concentrating hormone (MCH) neurons, which are intermixed with hypocretin cells in the normal brain, were not affected [121, 124], indicating that cell loss was relatively specific for hypocretin neurons. One study suggested gliosis, while the other found no clear evidence. Further studies using NARP and dynorphin, two markers colocalized with hypocretin in the posterior hypothalamus, also found decreased staining for these markers, indicating cell loss rather than lack of expression of hypocretin alone [125, 126]. Interestingly, two recent studies also found a compensatory increase in histaminergic cell number in the tuberomammilary nucleus in postmortem brains, indicating significant remodeling of wake-promoting systems following hypocretin cell loss [127, 128].

## Further HLA association and immunopathology studies in narcolepsy

The finding of hypocretin cell loss in narcolepsy, together with the demonstration that HLA-DQ0602 was mostly responsible for the association signal within the HLA region in the disease, rekindled the hypothesis of autoimmunity, with hypocretin cells as the logical target. Surprisingly, however, autoantibodies targeting hypocretin peptides were not found [129–131], and immunostaining of hypothalamic tissue with human narcolepsy sera did not reveal autoantibodies targeting co-localized antigens on these neurons [132–135].

Several red herring findings were made. Passive transfer experiments of human sera in mice have been published [136–138], suggesting the presence of functional autoantibodies with modulating effects on spontaneous colonic migrating motor complex contractions or reactions of rodent bladder strips to muscarinic stimulation, but we have tried and could not replicate the finding (data not shown). These investigators also suggested that peripheral injections of human narcolepsy sera in mice caused narcolepsy symptoms, but when we attempted replication, all had freezing seizures that may have been confused with narcolepsy events. Two of the 5 treated animals died, and hypocretin neurons were intact postmortem in all animals, including the 3 animals after recovery. Further, blinded sera samples were sent in Australia for the bladder strip assays, but opposite results to the initial findings were returned to us. Using a BAC-based transgenic animal model expressing a flag-tagged poly(A)-binding protein (*Pabpc1*) cDNA sequence in hypocretin neurons, Cvetkovic-Lopes et al. [139] isolated transcripts believed to be increased in hypocretin cells, including the protein Tribbles homologue 2 (Trib2). The authors went on to demonstrate increase Trib2 autoantibodies in recent onset narcolepsy cases, a result that was replicated by our group and a Japanese study using sera samples from subjects collected in the 1990–2000s, and some cross-reactivity of sera with hypocretin neurons.

Unfortunately, however, these authors may have been on the right track for the wrong reasons. Further studies using a similar approach but another mRNA-binding protein than Pabpc1, the protein P10, found that few of the genes expressed in hypocretin neurons as reported by Cvetkovic-Lopes et al. [139], including Trib2, were enriched in hypocretin neurons [140]. The result of the well-validated P10 technique was also confirmed by our own multiple expression array studies [141, 142]. Pursuing this line of investigation, we also found that TRIB2 autoantibodies were generally absent in more recent narcolepsy samples [143]. It is our hypothesis that TRIB2 autoantibodies may have marked a coinfection present together with a narcolepsy trigger in some cases with onset notably in the 1990s and 2000s, a result substantiated by the finding of a correlation between A/H1N1 and TRIB2 autoantibody levels in a recent study [144]. Interestingly, a recent study, reminiscent of the older Australian studies mentioned above, reported that local injections of purified immunoglobulins of narcolepsy-TRIB2-positive individuals but not controls, produced hypocretin cell lesions and narcolepsy symptoms [137]. Careful reading of this manuscript however does not support the conclusion of the study, as no hypocretin cell count statistics are provided, only an exemplar hypothalamic section showing widespread local cell loss that would be much larger than just hypocretin cell loss. Further, the authors report on “narcolepsy-like immobilization attacks” without associated EEG studies in 6 animals, which may well have been seizures considering their mean duration (66–464 s), much longer than typically reported in murine cataplexy (2–10 s) [120]. This brief discussion exemplifies the difficulties for others not in the field to make sense of a confusing literature. Only time will tell on whether or not the Tribbles story will hold on to scrutiny.

The absence of immunological findings led us to pursue the characterization of the HLA signal in narcolepsy. Sequencing studies of the HLA-DQ region, as well as studies of microsatellite markers in the region, indicated that no other gene was present in the susceptibility interval, and that the effect was in the DQ region [55, 57, 58]. Pursuing studies across multiple ethnic groups, a strikingly consistent pattern emerged. Indeed, not only was DQ0602 (the combination of DQA1*01:02 and DQB1*06:02), a near prerequisite for developing narcolepsy, but individuals homozygous for DQ0602 were at approximately 2–3 times greater risk of developing narcolepsy [95, 145–147], suggesting that the amount of DQ0602 heterodimer increased risk as well [148]. Intriguingly, we also found that DQ0602/DQB1*03:01 were also at increased risk versus other combinations [95, 146, 147], an effect difficult to explain as it occurred in the context of multiple DQα-associated alleles (DQA1*03:01, DQA1*03:02, DQA1*05:05 and DQA1*06:01), suggesting it was not mediated via a DQα/β heterodimers. This effect was confirmed in trios using transmission disequilibrium tests, a design where power is enhanced by the removal of alleles that are located together with DQ0602 in DQ0602-positive parents and thus never transmitted [149].

**Fig. 5:**
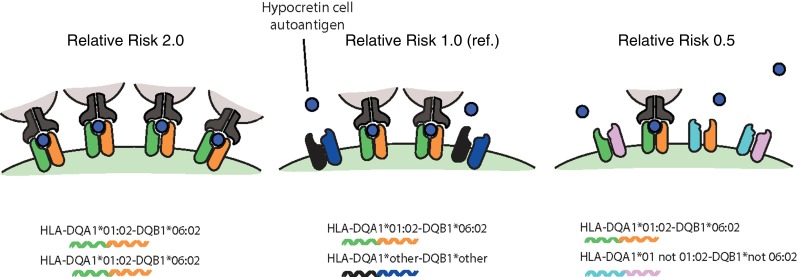
Allele competition model explaining HLA-DQ effects in narcolepsy. HLA-DQB10602 is almost a prerequisite for developing narcolepsy, probably because it can binds the culprit HCRT epitope. In addition, consistent effects are observed across multiple studies, with HLA-DQ0602 dosage/amount influencing risk of developing the disease. For example, subjects homozygous for DQ0602 have twofold higher risk of developing narcolepsy in comparison with most DQ0602 heterozygotes. At the opposite, DQ0602 heterozygotes that have other DQ1 alleles *in trans* that can heterodimerize with the DQα and DQβ alleles of DQB0602 have a twice lower risk, as predicted from the allele competition model [95, 145, 147]

In addition to this effect, protective effects of DQB1*05:01, DQB1*06:01, DQB1*06:03, and other DQ1 alleles that are non-DQ0602 were found [95, 146, 147, 149–153]. DQ1 is a broad DQ subtype that includes the DQα alleles encoded by DQA1*01 and DQβ alleles encoded by DQB1*05 and 06 subtypes. These DQ1 alleles, unlike those of the other broad DQ groups (DQ2, 3, and 4), are “compatible” with each other, meaning that they have sequence similarity and proper folding as selected by invariant chain binding (in contrast to non-DQ1 subtypes such as DQ2 and DQ3 are generally compatible with each other). Estimating relative risk, we noted that risk of DQ0602/other DQ1 was about one-half of DQ0602/other, indeed suggesting that there is competition of transencoded DQ1 alleles that are non-DQ0602, reducing the amount of DQ0602, and thus risk, a phenomenon we called allele competition [95, 153] (Fig. 5).

## Genome-wide association studies (GWAS) in human narcolepsy indicate association with T-cell receptor loci (TCR) and other autoimmune associated loci

Whereas the decades spanning 1985–2005 saw the emergence of positional cloning as a powerful tool to isolate highly penetrant disease genes, the release of the first complete human genome sequence in the early 2000 led in 2005 to more systematic, large-scale genome-wide association studies (GWAS) where up to a million single-nucleotide polymorphisms can be tested at once in a subject. This allowed investigators to better describe the genetic architecture underlying multigenic disorders. Using this technique in narcolepsy in 2009, we found that the disease was strongly associated not only with HLA, but also with a specific polymorphism in the TCR alpha gene [154]. Although genetic risk was not high (OR ~ 2) when compared to effects found with HLA polymorphisms, the finding was nonetheless remarkable as it further demonstrated a role of the immune system in narcolepsy. It was also unusual, as none of the other autoimmune disorders that have been subjected to GWAS analysis have TCR loci as susceptibility factor.

Further studies in larger and larger samples that also included other ethnic groups, notably Chinese, Japanese, and African Americans, were conducted and led to the identification of other associated genes, most known to be involved in other autoimmune diseases [155–157]. Other associated loci included the TCR beta-gene a partner of TCRα; TNFSF4 (also called OX40L) a costimulatory receptor for T-cell activation involved in Lupus [158], Crohn’s disease [159], rheumatoid arthritis [160], and celiac disease; Cathepsin H, an enzyme likely involved in antigen processing and associated with Type 1 diabetes [161]; ZNF365, a transcription factor associated with inflammatory bowel disease (IBD) [159] and atopic dermatitis [162]; IL10RB-IFNAR1, a region associated with IBD [163].

Of additional interest was the finding of an association within the PPAN-P2RY11-EIF3G gene region, 10 kb from the DNA methylase gene 1 (DNMT1) [156]. This finding was notable as this gene region was not known to be associated with other autoimmune diseases, although P2RY11, an ATP receptor, regulates cell death, notably in immune cell subsets. Interestingly, in a parallel exome sequencing project of rare dominant phenotypes with narcolepsy, we found that a rare disease associating late-onset narcolepsy with deafness, cerebellar ataxia, and dementia (ADCA–DN) was secondary to mutation in exon 21 of the DNMT1 gene, resulting in late-onset neurodegeneration, with a likely effect on hypocretin cells [164]. Further mapping of the GWAS signal confirmed location within P2RY11-EIF3G and not extending to DNMT1, although regulatory elements for the latter could still lie within the nearby region. In favor of this hypothesis, although P2RY11 is a pseudogene in rodent, the syntenic block containing PPAN-P2RY11-EIF3G-DNMT1 synteny is conserved from zebrafish through mammals.

Overall, these GWAS association studies did not reveal any smoking gun evidence, but strongly confirmed that the etiology of narcolepsy was likely autoimmune. A more detailed analysis of the pathway suggested a primary importance of HLA-DQ0602 presentation to CD4^+^ cells and T-cell mediation.

## Rare HLA-DQB1*06:02-negative subjects with primary narcolepsy

The issue of whether or not HLA-negative subjects with narcolepsy had a true disease, i.e., hypocretin deficiency, has been debated since the discovery of the HLA-DR2 association in narcolepsy. The discovery that DQB1*06:02 was a better marker than DR2, notably in African Americans, helped resolved some of this debate. Similarly, one DQB1*06:02-negative subject with low CSF hypocretin and very early onset (6 months) is likely secondary to a damaging hypocretin mutation in its signal peptide, but these findings still left a few unexplained cases with usual childhood or adolescent onset, hypocretin deficiency, and DQB1*06:02 negativity.

To further our understanding of these exceptionally rare cases, which we estimate represent approximately 2 % of cases and vary in frequency across countries (maybe more frequent in Italy), we further characterized 8 such cases with documented low CSF hypocretin through exome sequencing and full HLA typing. Interestingly, we found that 4 of 8 cases carried DPB1*09:01, a subtype that should have been rare in this multiethnic sample (~5 %) [165]. This result suggests that another heterodimer, possibly DPA1*0201/DPB1*09:01, may also play a role in exceptionally rare cases of autoimmune hypocretin deficiency.

## The role of upper airway infections in triggering narcolepsy

Starting in the mid-2000s, narcolepsy became increasingly recognized, giving us the opportunity to study patients closer to onset, thanks to faster diagnosis [166]. Blood samples could also be collected closer to onset for biological analysis. In particular, we saw more and more young children with a recent onset, and as these cases often have an explosive and rapid evolution, we were able to query circumstances surrounding onset. We noted a number of reports of a past history of streptococcus infections (i.e., strep throat), which resulted in tonsillectomy and hospitalization in some cases, and in one case with suspected pediatric autoimmune neuropsychiatric disorders associated with streptococci (PANDAS) [167]. Streptococcus infections were interesting as they are known to be associated with onset of rheumatic heart fever, Syndenham’s Chorea [168], two other autoimmune diseases (although these have decreased in frequency in the western world with the use of antibiotics), and PANDAS, a more controversial psychiatric entity. Further, older studies had suggested an association of narcolepsy with antistreptolysin-O (ASO) and anti DNAse B, two markers of recent streptococcus infection, although this was not replicated [169–171]. Intriguingly, increased ASO has also been reported in recent cases of encephalitis lethargica [172], which still occur today at low frequency (although differential diagnosis with anti-NMDA encephalitis may be difficult).

Still searching for the elusive autoantigen, we conducted Western blot studies of selected rat brain regions, stained with narcolepsy sera, and noted a frequent pattern of cross-reactivity with a 58-kDa protein in many samples [173]. In parallel with this work, we decided to re-evaluate whether recent narcolepsy samples had increased titers of ASO, arguing that past studies may have been variable because distance to onset was not controlled [169–171]. Antibodies against Helicobacter pylori [Anti Hp IgG] were also tested, as this infection had been suggested to be involved in the triggering of idiopathic thrombocytopenic purpura, another autoimmune disease. To our surprise, we found that high ASO titers were found more frequently in patients within 1 or 3 years of onset, compared to age-matched controls or patients with long-standing disease [174]. This finding was interesting as a parallel epidemiological study also found increased risk of developing narcolepsy when reporting past streptococcus infections [175]. In additional studies, we also discovered that the 58-kDa cross-reactive protein is protein disulfide isomerase (PDI), an abundant protein with pleiotropic metabolic, immunologic, and thrombotic effects [173, 176].

**Fig. 6:**
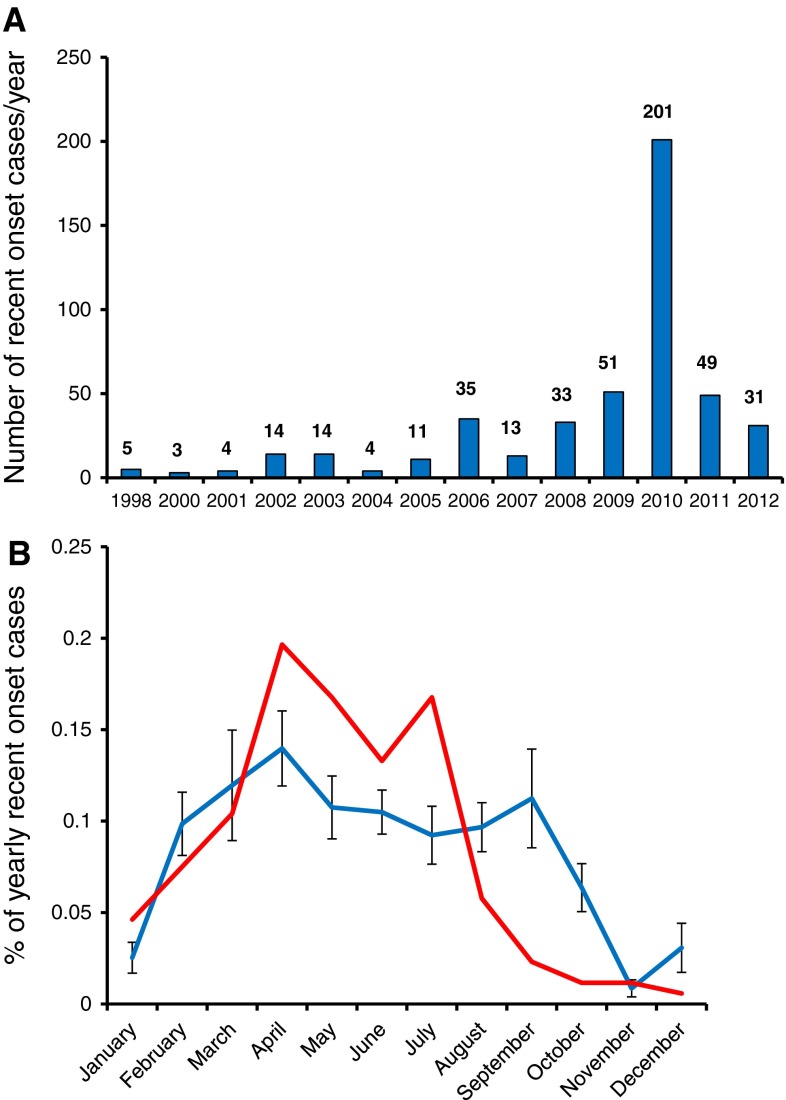
**a** Yearly occurrence of recent onset (onset within a year of onset) showing a dramatic increase in 2010, following the H1N1 pandemic of 2009, with return to baseline condition the following years [178, 184]. **b** Seasonal pattern of onset of narcolepsy in Chinese narcoleptic patients showing highly increased risk in spring and summer versus early winter

In parallel with this work, starting in 2000, a strong collaboration between Stanford and Beijing University was established. In 2004, as the world federation for sleep society met in Zuhai, publicity surrounding narcolepsy led to increased case recognition in China and referral to the Beijing University sleep center. Interestingly, the large majority of cases diagnosed at the center in Beijing were children (70 %) [177], many with abrupt onset, a pattern that we attributed to increased ascertainment and vigilance due to the one child policy in China; many more adults, approximately 400,000 Chinese narcolepsy subjects, should be present in the general population of this country based on the established prevalence. As onset in these children was extremely clear and could be generally dated to the exact month if not week by parents, we decided to examine whether a seasonal pattern of onset was present, as would be predicted based on our suspicion of an association with upper airway infections. Strikingly, we found that onset was about 6 times more frequent in late spring versus early winter [178], consistent with the hypothesis that most cases of narcolepsy were triggered by winter upper airway infections (Fig. 6).

## The Pandemrix and pandemic 2009 H1N1 influenza tragedy

In the spring of 2010, a number of events converged to indicate that a specific trigger, likely the 2009 H1N1 pandemic influenza, had a significant effect in increasing onset of narcolepsy in young children. To review the background, in the spring of 2009, a new strain of influenza A H1N1 of likely swine origin appeared in Mexico, spreading rapidly in humans, and affecting young adults with a high reported case fatality rate of 0.4 % [179]. This caused alarm, as with such a high mortality rates, millions of death were likely worldwide when the new virus would hit the world population the following winter. Faced with such a threat, the World Health Organization (WHO) and other organizations encouraged vaccine makers to initiate large-scale production of vaccines targeting the new strain, which had not been included in the 2009–2010 regular trivalent season flu vaccine [180]. To generate these vaccines, almost all manufacturers used a A/*California*/7/2009 (*H1N1*)-pdm09-like reassortant virus containing hemagglutinin HA type 1, neuraminidase NA type 1 (thus H1N1), and polymerase basic 1 (PB1) proteins from A/California*/*7/2009, on a backbone H1N1 virus PR8, derived from an older, A/*Puerto Rico/8*/1934, H1N1 virus [181].

As predicted, p 2009 H1N1 spread rapidly and became the dominant influenza strain the following winter. Fortunately, however, mortality was not as high as anticipated, ranging closer to that of a regular seasonal flu [182, 183]. Soon after, in the spring of 2010, we noted that a much higher number of children with recent onset were referred to our center when compared to prior years [143]. Further, in China, the 2010 spring and summer were exceptional, documenting a 3–5 times increase in the number of children with narcolepsy when compared to prior years, a peak that appeared 4–6 months after the peak of H1N1 infections [178, 184].

In parallel with this and perhaps most strikingly, in both Finland [185, 186] and Sweden [187–189], cases of childhood onset narcolepsy were reported a few months following vaccination with a particular pH1N1 vaccine formulation called Pandemrix, documenting a ~tenfold increased risk of developing narcolepsy following vaccination [143]. Other studies confirmed that this particular vaccine had similar effects in Norway [190], England [191], France [192], and Ireland [193, 194], although it is important to realize that only ~1/15,000 children vaccinated with Pandemrix ever develop narcolepsy (including DQ0602 siblings and in at least one case a discordant twin).

Pandemrix is a unique vaccine, manufactured in Dresden by Glaxosmithkline (GSK) using a Fluarix manufacturing process to isolate surface antigens (typically purifying mostly the HA protein, which is dosed at 3.75 µg H1 in this vaccine) [181, 193, 195]. In addition, a specific adjuvant, AS03A, a mix of squalene (10.68 mg), DL-α-tocopherol (11.86 mg), and polysorbate 80 (4.85 mg), was added. The AS03A adjuvant is potent at stimulating CD4^+^ T-cell responses [196], and it is clear that vaccine efficacy was high; only one injection found to be sufficient to obtain high coverage as measured by the hemagglutinin inhibition assay notably when geometric mean titer are compared to other vaccines (an assay measuring antibodies targeting the HA protein) [181].

Other vaccines that have been used were manufactured using different protocols to isolate surface antigens and/or different adjuvants. Arepandrix, a vaccine also produced by GSK but in Laval, Quebec, is identical to Pandemrix, except that a slightly different process of isolation of surface antigens (the Flulaval process) was used [193, 195]. Focetria, a Novartis vaccine, is another vaccine relatively similar to Pandemrix. It uses a MF59 adjuvanted, containing 9.75 mg of squalene and 7.5 µg of H1 and 1.175 mg of polysorbate, and contains a more pure H1 preparation [181, 193, 195]. Arepandrix, which has been used in Canada, has recently been found to increase the risk of narcolepsy, but more weakly, 1.5- to 3-fold [197]. Although no study has been formally done, Focetria has not been reported to trigger many cases of narcolepsy.

In the United States, only non-adjuvanted or live attenuated vaccines have been used. Of interest is the fact all seasonal trivalent split or subunit vaccines that have been used since 2009 still contain A/*California*/7/2009 (*H1N1*)-pdm09-like reassortant as one of the three strains covered. Although this has not been formally studied and sporadic cases have been reported, the effect on narcolepsy risk for these vaccines is likely either protective, inexistent, or weakly predisposing. Certainly, no strong signal has been reported to cause alarm.

In summary, it appears that in the spring and summer of 2009, a larger than usual number of childhood cases was observed in China and probably in other countries independent of any vaccination. In addition, cases of narcolepsy in children also occurred in reaction to Pandemrix, although overall risk was small. The effects of other pH1N1 vaccines were either much milder or nonexistent.

## Hypocretin as the culprit autoantigen and demonstration of CD4^+^ T-cells reactive to hypocretin when presented by DQ0602

In view of our genome-wide association data indicating T-cell receptor associations and the lack of detectable autoantibodies in serum, we next focused our investigations on T-cell reactivity, starting with hypocretin (HCRT) as the possible culprit autoantigen. We elected to use enzyme-linked immunoSpot (ELISpots) as the technique of choice as it is one the most sensitive tests to detect rare autoreactive T-cell clones. This test measures the activations of T cells by antigens presented by antigen-presenting cells (APCs) through the local trapping of secreted cytokines on antibody-coated wells, creating “spots” that can be revealed every time a cell is activated. Prior unpublished studies in our laboratory used this test with full hypocretins 1/2 sequences and peripheral blood mononuclear cells (PBMCs) or dendritic cells as APCs had not been successful (unpublished data), showing multiple spots and no differentiation between control and narcolepsy.

We thus decided to increase the specificity by separating CD4^+^ and CD8^+^ T cells, smaller peptide fragments, and APCs carrying only HLA-DQ0602. To create a DQ0602-specific APC cell line, Mellins et al. transfected T2 cells lacking the expression of both MHC class I and class II with DQA1*01:02 and DQB1*06:02, creating a T2.DQ602 cell line [198]. We then used purified HLA-DQ0602 and biotin-labeled EBV_490–503_ as a known ligand, to scan overlapping 15 mers covering the entire preprohypocretin peptide for binding. Using this technique, we could identify and test a total of 10 core sequences with binding to DQ0602 for presentation to CD4^+^ T cells. Additional experiments used more classic, autologous monocyte-derived dendritic cells as APCs were also performed, starting with CD4^+^ T cells since those are those recognizing HLA class II molecules such as DQ0602 [198].

**Fig. 7:**
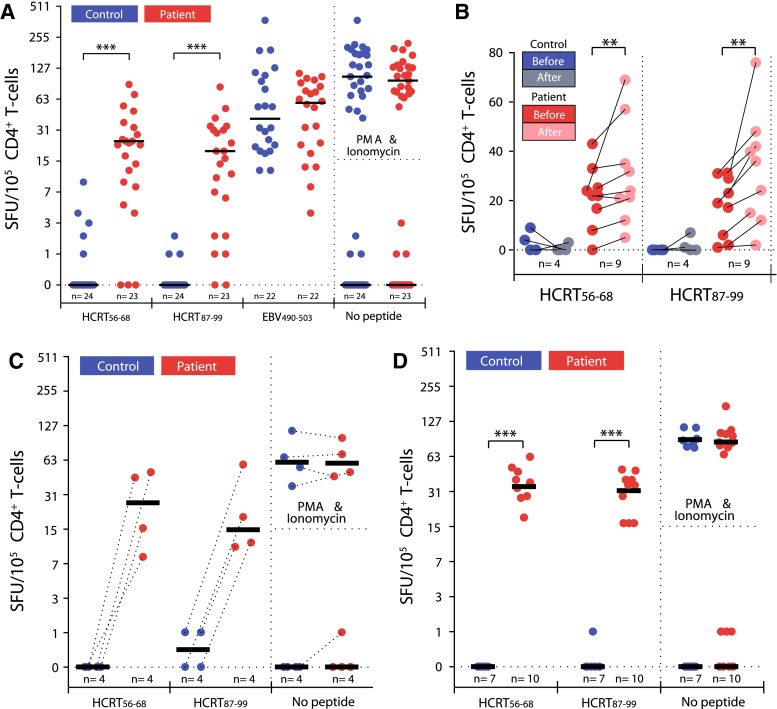
CD4^+^ T-cell reactivity to HCRT_56–68_ and HCRT_87–99_ as presented by DQB0602 in narcolepsy (*red dots*) but not DQ0602 controls (*blue dots*), in comparison with pHA1_275–287_. **a** Random DQB0602 controls and patients. **b** Effects of a seasonal vaccination with a trivalent, non-adjuvanted influenza vaccine containing pH1N1, H3N2, and influenza B. **c** Comparison of 4 discordant monozygotic twin pairs. **d** Comparison of Pandemrix vaccinated DQB0602 siblings with and without narcolepsy. Data obtained using enzyme-linked immunoSpot (ELISpot) from De la Herran-Arita et al. [198]

Intriguingly, in most cases, HCRT binders presented to DQ0602 produced CD4^+^ T-cell reactivity in both controls and patients, a finding we believe may not occur in vivo, for example because these hypocretin peptide fragments may never be processed by APC for presentation [198]. For three binders, however, no reactivity was recorded in controls, suggesting that T cells reactive to these fragments are either anergized or absent, probably as a result of tolerance. One of these fragments was HCRT_1–13_, a known signal peptide binder that had been crystalized with DQ0602 [199]. The two other fragments were homologous C-terminal end regions of the secreted hypocretin 1 and hypocretin 2 peptides, HCRT_56–68_ and HCRT_87–99_, regions required for the activation of hypocretin receptors [200]. When presented to narcolepsy versus control CD4^+^ T cells, a differential activation in narcolepsy but not controls was found with HCRT_56–68_ and HCRT_87–99_, suggesting these may be involved in the pathophysiology of hypocretin cell loss in narcolepsy. C-amidated, functional fragments of the secreted hypocretin 1 and hypocretin 2 peptides were also able to produce the same effect [198]. A total of approximately 50 patients and 50 DQ0602 controls were finally tested with the same antigens, including discordant monozygotic twins and siblings vaccinated with Pandemrix with and without narcolepsy. In all cases, the test predicted narcolepsy with high specificity (100 %) and sensitivity (~90 %), indicating diagnostic value (Fig. 7). Using other cytokines, we also found the T-cell response to be consistent with a Th1 and Th17 response, as usually found in other autoimmune diseases [198].

## Identification of a H1 2009 flu peptide as a molecular mimic of hypocretin

**Fig. 8:**
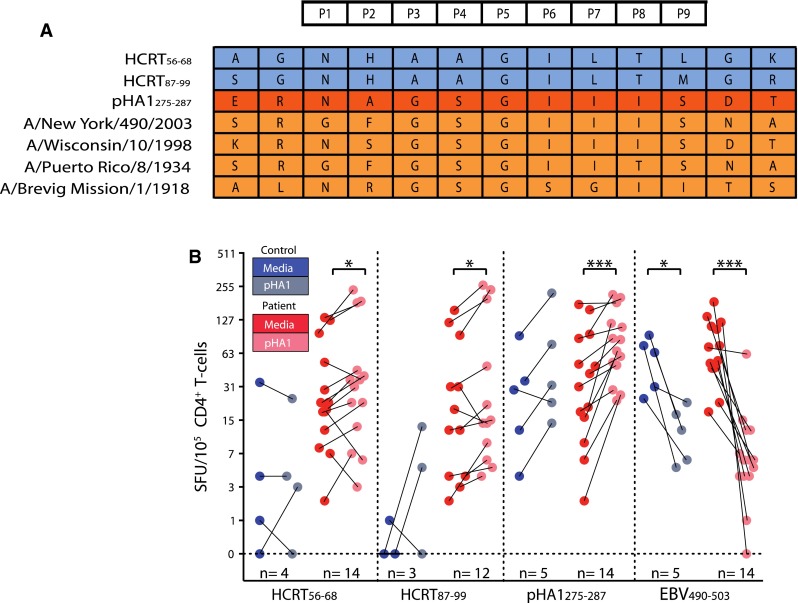
pHA1_275–287_ as a mimic of HCRT_56–68_ HCRT_87–99_. **a** Alignments of HCRT_56–68_ with pHA1_275–287_ and other HA sequences, showing similarity. **b** Cultures of narcolepsy T cells with pHA1_275–287_ increases hypocretin-autoreactive cells [198]

Because data suggest narcolepsy may be triggered by pH1N1 infections or vaccinations, we next hypothesized that fragments specific of the pH1N1 2009 virus, also contained in vaccines, could have homology with HCRT_56–68_ and HCRT_87–99_, resulting in molecular mimicry. Considering that only the hemagglutinin H1, the neuraminidase N1, and polymerase basic protein 1 PB1 proteins of influenza A are present in the pandemic vaccines and wild-type infections, we screened these proteins for binders to DQ0602, as previously done for preprohypocretin. Among a total of 31 strong and 76 weak binders representing approximately 55 epitopes (15 uniques) found in these proteins, we rapidly realized that pHA1_275–287_ was unusual, as it was partially homologous to HCRT_56–68_ and HCRT_87–99_, and had a sequence specific for the pH1N1 2009 strain. Further, amino acids important for HCRT_56–68_ and HCRT_87–99_ binding to DQ0602 and TCR activation as defined by substitution screens were conserved in the potential pHA1_275–287_ mimic (Fig. 8).

To test whether mimics of HCRT_56–68_ and HCRT_87–99_ were present in Pandemrix, we already had used vaccine protein extracts from Pandemrix and found that hypocretin CD4^+^ T cells of patients but not controls grew more numerous in 10-day cultures in the presence of these extracts [198]. This result indicated that something in the vaccine could cross-stimulate hypocretin-autoreactive T cells, as predicted if molecular mimicry occurred. Other experiments with acute presentation of vaccine proteins by T2.DQ602 cell lines and isolation of activated patient CD4^+^ T cells through capture of CD4 cells positive with CD38 also led to enrichment of hypocretin-autoreactive T cells, in agreement with this hypothesis. Our next step was thus to test whether pHA1_275–287_ could be the culprit behind this effect. To test this hypothesis, we repeated the cross-culture and CD38 capture experiments with pHA1_275–287_ and found that, indeed, the peptide was able to produce the same effect, although in general the effect was weaker than with the entire vaccine extract [198]. Although molecular mimicry with infectious agents has long been suspected as a possible trigger for autoimmunity [201–203], the H1N1–narcolepsy association may be the clearest instance of such a phenomenon [195].

## Mediation of hypocretin cell killing

Although our results explain much, and may be the first clear demonstration of molecular mimicry in humans, several questions remain unanswered. First and foremost, CD4^+^ T cells are not likely to be the ultimate cell population causing hypocretin cell destruction. In most other cases, CD4^+^ T cells are acting as helper T cells, directing antibody formation by B cells and coordinating a CD8^+^ T-cell response that is cytotoxic and creates the cell destruction. In narcolepsy, we found no evidence of antibody involvement, a phenomenon we hypothesize is due to the fact hypocretin is specific to the brain. In the peripheral circulation, B cell help through class II presentation of antigens directs the production of antibodies. It may be that hypocretin presentation by HLA DQB0602 on B cells to autoreactive CD4 cells does not occur because B cells are not commonly present in the central nervous system unless the blood–brain barrier is very much disrupted. Although B cells can traffic to the brain through the integrin system as T cells, it is unclear whether this occurs under normal conditions, as most of the studies supporting this have been done in patients with multiple sclerosis. If this hypothesis is correct, autoimmune diseases directed against brain-specific antigens may have less B-cell involvement, providing there is no blood–brain barrier breakdown.

A more likely hypothesis may be the involvement of a CD8^+^ T cytotoxic cell population, although it is possible CD4^+^ T cells themselves or a novel mechanism specific to the brain are involved, for example involving natural Killer cells or microglia phagocytosis. Whatever the mechanism should be, the specificity of the cell loss likely involves the recognition of antigens specific of hypocretin cells. It is notable that our CD4^+^ T-reactive cells can recognize truncated fragment of the secreted neurotransmitter [198], a product available broadly in the interstitial field. Local concentration gradients of hypocretin, other epitopes, or other antigens could possibly play a role. Further studies in this direction is likely to teach us much on how the immune system treats neurons, a population of cells that is particular as it cannot express HLA class II and is not easily replaceable.

## T-cell receptors involved and other mimics

A key experimental next step toward our understanding of narcolepsy is the identification of the culprit T-cell receptors (TCRs) cross-reactive with hypocretin on CD4^+^ T cells. As mentioned above, genetic associations with TCR polymorphisms have been found, notably within the J24 segment [154, 157]. It is thus likely that J24 containing recombinant TCRA sequences are involved in disease susceptibility, suggesting that the TCR response involved in the disease may not be as polyclonal as for other autoimmune diseases, also explaining the association signal in TCRB. If this is correct, it will greatly facilitate our understanding of the TCR-mediated CD4^+^ T-cell response, although it is possible that the TCR genetic effects are mediated through CD8^+^ T-cell recognition. To achieve the isolation of culprit TCRs, we are using DQ0602-HCRT_56–68_/HCRT_87–99_ and DQ0602-pHA1_275–287_ tetramers, culture/cloning enrichment and single cell FACS sorting followed by sequencing of individual T cells. Additional work involves whole repertoire TCR next-generation sequencing, cleaning of sequencing errors, followed by bioinformatics allowing pairing of alpha- and beta-chains.

Based on our current data, we suspect that only a subpopulation of pHA1_275–287-_positive cells will cross-react with hypocretin, suggesting that either pHA1_275–287_ is only one of several mimics, and/or that hypocretin-specific, non-H1N1 cross-reacting T-cell populations are subsequently selected following the initial mimicry events. Understanding how the TCR response toward hypocretin and various mimics of various affinities differs in complexity and number of distinct receptors will likely be a topic of great interest for our understanding of molecular mimicry. In addition, crystallography will also answer the question on whether or not hypocretin receptors bind abnormally/atypically to the TCR complex, as reported for most other cross-reactive TCRs that have been isolated in other diseases. As cross-reactivity of T-cell receptors is now believed to be the norm rather than the exception, we believe it is likely a hierarchy of mimics of various affinities could be involved. In this context, experiments in animal models will be needed to establish local concentration of mimics in physiological conditions. Identifying mimics that were involved prior to 2009 will also shed additional light on this important question. It is possible that viral genetic diversity, the abundance of particles upon infection, and neurotropism make influenza important in the shaping of the immune system and the triggering of narcolepsy and perhaps other autoimmune diseases.

## Breakdown of immune tolerance

**Fig. 9:**
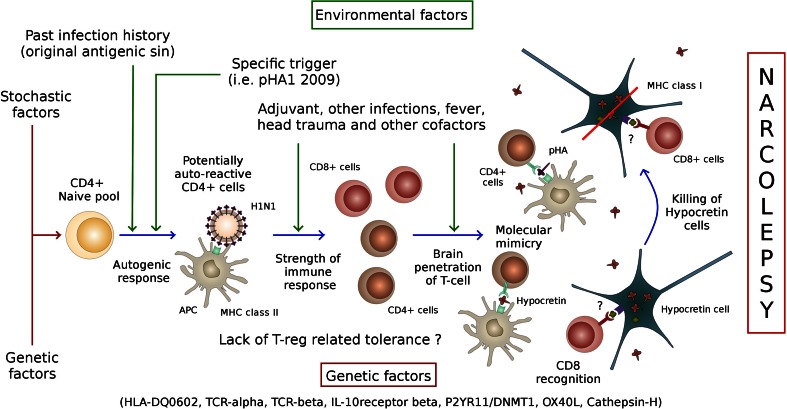
Pathophysiological model for narcolepsy and road map for future experiments. Narcolepsy may result from the succession of unlikely events, starting with a predisposing genetic background, stochastic events leading to the generation of potential pathogenic naïve T cells, inadequate central tolerance, cross-stimulation with various environmental mimics at the wrong developmental time (in relation to other prior infections), the absence of peripheral tolerance, penetration in the central nervous system of culprit T cells, molecular mimicry, and destruction of hypocretin neurons by CD8^+^ T cells or other mechanisms. Other epitopes may also be involved, notably those implicating CD8^+^ T cells

Another important question in the field of autoimmune disease is how and why is tolerance toward autoantigens brakes. The fact one of our polymorphism is located close to the IL10RB suggests the importance of inhibitory signals [204]. Whether or not hypocretin is expressed in the thymus is unknown, and examining the frequency of naïve cells cross-reactive to hypocretin epitopes may shed light on the role of central tolerance. Similarly, regulatory T cells could be involved and, if identified, corresponding TCRs were sequenced and compared to autoreactive TCRs. It is of interest that in many of our culture conditions notably with PBMCs, enrichment of hypocretin cross-reactive cells seems more difficult when the cells are cultured with hypocretin in comparison with the pHA1_275–287_ mimic. Figure 9 describes a potential roadmap to study the pathophysiology of narcolepsy.

## Why did Pandemrix have such a strong effect in Northern Europe?

The finding of a mimic inside the H1 protein, pHA1_275–287_, does not entirely explain why Pandemrix, as opposed to other Pandemic vaccines, was particularly potent at triggering narcolepsy. Two major explanations could be involved, differences in vaccine composition or in the target population of vaccinees [195, 198]. With respect to vaccine composition differences, HA is the main protein dosed in all vaccines and reported as reference for comparison; other viral components are present in lower amounts [181, 193]. A several other vaccines have a higher concentration of HA by dose than Pandemrix, it is surprising that HA would be the culprit.

One possible explanation could be the effect of the AS03A adjuvant at promoting CD4^+^ T-cell response [196], as it is possible this adjuvant is more potent than MF59 (squalene) alone [181], or has more effect on blood–brain barrier permeability. This explanation however does not explain why Arepandrix was weaker than Pandemrix at increasing narcolepsy risk (OR = 1.5–3) [197], as it contained the same AS03A adjuvant and presumably the same HA concentration. Interestingly, however, as the antigen preparation protocol is different between Pandemrix (Fluarix protocol), Arepandrix (Flulaval protocol), and Focetria (Agrippal Protocol) [205], additional mimics on proteins others than HA could be involved (i.e., NA, PB1, or less probably antigens in the backbone that could work in conjunction with pHA1_275–287_). Prior studies have clearly shown these proteins to be differently purified in these protocols, with the Agrippa process producing more pure HA than the fluarix process [205] (no information is available on the flulaval process). It is also possible that the various inactivation processes used in these protocols, or differences in excipients, produce differential antigenic modification (oxidation, etc.) that could play a role. We are pursuing these hypotheses.

Other factors not related to the vaccine itself could also have played a role. Indeed, even in children and with Pandemrix, absolute risk remains low, 1/15,000 vaccinees, so that many other factors (environmental, stochastic, or genetic) in addition to Pandemrix are likely to be needed for a subject to develop narcolepsy. As the various vaccines have been used in different populations, population-specific differences could make it seem like various vaccines have different effects. One possible difference could involve genetic polymorphisms enriched in Northern Europe. In a recent study, we found evidence for a genome-wide significant difference in a SNP located between the DR and DQ genes when comparing Chinese patients who developed narcolepsy prior versus after September 2009 [157]. Nonetheless, odds ratio for such a polymorphism remained low, and there is no evidence of strong population differences for this SNP across Caucasians.

A second aspect could involve coinfections and timing of vaccination in relation to how the H1N1 pandemic itself unfolded in various countries. Prior data suggest that other infections, such as streptococcus, may have played an additional role in some population [174, 175], although it could just be a secondary association [206]. Regarding H1N1 infection by themselves (a factor known to increase risk based on our Chinese data [178, 184] ), peak vaccination occurred 2–3 weeks following the major wave of infection in most of the signal countries [207]; thus, a double hit could have played a role in either direction (protection or adding predisposition by increasing the immune response).

To address this issue, a virus-specific NS1 B-cell epitope not present in the vaccine backbone was identified and antibodies directed against it evaluated in Western blots [208]. As reactivity was only found in subjects after documented natural infections but not in post-Pandemrix cases, the authors concluded a double hit is unlikely. Although tantalizing, we believe that exploring only one epitope is not sufficient to conclude, considering HLA dependence of these effects. It is also possible that the narcolepsy immune response may not implicate the NS1 epitope in infected subjects because of immunodominance differences in the response. Interestingly, preliminary data in Sweden suggest that Pandemrix had less effects on narcolepsy in subjects in the North of the country, where infections came before the vaccination, possibly suggesting that infections could have been protective. In summary, the potential role of coinfections is uncertain especially considering the fact about the same timing of vaccination in relation to natural pandemic infection occurred in Canada with Arepandrix and in Scandinavia with Arepandrix. Nonetheless, Focetria was more used in Southern Europe, for example in Italy, when H1N1 infections likely reached epidemic proportion later in the year. A third possibility explaining variability in response to the various adjuvanted vaccines is target population and how many adults versus children were vaccinated and in what sequence [181, 207].

Finally, and maybe most importantly, these populations are all distinct in their past immunological history and original antigenic sin is likely to have played a role. As young children were mostly at risk, it is possible that past exposure to other prior influenza strain or other infections mitigates risk, maybe simply because in these cases, a reactivation of memory cells that are targeting a prior immunodominant peptide common to prior strains reduces the probability of engaging T cells specific of new mimics present in pH1N1 in a naïve immune response. Alternatively, new epitopes could reengage a population of memory cells that have seen a prior antigen and can be cross-reactive. We believe that the study of cross-reactive T-cell populations in naïve and memory compartments of controls of various origin and age, for example with hypocretin tetramers, will answers some of these questions.

## Conclusion

Since my arrival at Stanford in the late 1980s, I have been blessed with the unique collaborative ambience of the University and its immunology program. Trained as a molecular pharmacologist and a psychiatrist, I was lucky to find collaborators such as F. Carl Grumet at the Blood Bank who, like me, thought that positionally cloning the canine narcolepsy gene was not only possible but also the logical next step. Collaborators such as Lucas Cavalli Sforza in genetics and Joachim Hallmayer open my eyes to the power of human genetics. Moving into immunology, I was exposed to the best and the brightest, starting with deep HLA and immunogenetic training, and then to general immunology with Mark Davis, Carl Grumet, Hugh Mac Devitt, Betsy Mellins, Larry Steinman, and others. The small size of the University, the accessibility of its leader, the excellence and diversity of its faculty, and a willingness to dare and collaborate outside of your field and comfort zone is what made Stanford unique, notably in immunology.

Our current results indicate that narcolepsy, a disease caused by hypocretin cell loss, is associated with autoreactive CD4^+^ T cells recognizing fragments of hypocretin when presented by DQ0602, an HLA allele strongly associated with the disease. As the cause of the symptoms is the loss of hypocretin, we believe this population of autoreactive CD4^+^ T cells is very likely within the causal pathway for narcolepsy. Influenza A, notably 2009 pH1N1, is a likely environmental trigger of the autoreactive CD4^+^ responses. Using two independent techniques, we found that Pandemrix pH1N1 viral extracts were able to activate hypocretin-autoreactive cells, suggesting that molecular mimics were present in the vaccine and the wild-type virus. One particular mimic, pHA1_275–287_, was identified within the virus as a likely culprit for the cross-reactivity. Together with other data, these findings suggest that influenza can be the trigger of autoimmune responses, notably narcolepsy.

In the future, we plan to explore how hypocretin cells are being destroyed, study where and why central and peripheral tolerance toward hypocretin is broken in the disease, identify other potential mimics and explore population- and vaccine-specific effects that may be involved in precipitating narcolepsy. Isolation and characterization of culprit T cell is ongoing to demonstrate mimicry. Eventually, animal models will be needed to demonstrate pathological effects of specific T-cell clones so that Koch’s criteria of causation are met [203].

Additional work will include the study of cases without cataplexy or of mild hypersomnia cases present in the population, and immune therapeutic attempts in subjects identified before the hypocretin cell loss is complete and irreversible. Non-open-labeled studies using IVIg have produced mixed results [209–214], not surprisingly considering the absence of current evidence for B-cell involvement. We believe that these studies will teach us much regarding vaccine safety and autoimmunity in general, notably when it is directed against neurons. A likely advantage of narcolepsy as a model for autoimmunity is it relative simplicity: the involvement of a single primary HLA heterodimer, possibly a single CD4^+^ T-cell epitope sequence derived from secreted hypocretin peptides, and a likely relatively focused, oligoclonal causative T-cell response.

In parallel with immune-related work, the expected introduction of hypocretin antagonists (called dual orexin receptor antagonists, DORA) as hypnotics will revive interest in this target for drug development. Hypocretin does not cross the blood–brain barrier well, and although creating brain penetrating agonists for peptide receptor can be challenging, it is likely a matter of time before such compounds become available for treating daytime sleepiness and narcolepsy.
